# Metabolic Potential of *Candidatus* Saccharimonadia Including Rare Lineages in Activated Sludge

**DOI:** 10.1111/1758-2229.70231

**Published:** 2025-11-06

**Authors:** Shuka Kagemasa, Kyohei Kuroda, Ryosuke Nakai, Mikiko Sato, Yu‐You Li, Kengo Kubota

**Affiliations:** ^1^ Department of Civil and Environmental Engineering Tohoku University Sendai Japan; ^2^ Department of Creative Technology Engineering National Institute of Technology, Anan College Tokushima Japan; ^3^ Biomanufacturing Process Research Center National Institute of Advanced Industrial Science and Technology (AIST) Sapporo Japan; ^4^ Department of Frontier Sciences for Advanced Environment Tohoku University Sendai Japan

**Keywords:** activated sludge, *Candidatus* Saccharimonadia, metagenome analysis, *Minisyncoccota*, size fractionation, type IV secretion system

## Abstract

*Candidatus* Saccharimonadia is a class‐level lineage of ultrasmall bacteria within the phylum *Minisyncoccota* (formerly Candidate Phyla Radiation or *Ca*. Patescibacteria), commonly found in activated sludge processes treating municipal wastewater. In this study, we aimed to elucidate the metabolic potential of *Ca*. Saccharimonadia by using shotgun metagenomic sequencing combined with a filtration‐based size‐fractionation approach for activated sludge from five wastewater treatment plants. A total of 65 high‐quality metagenomic bins were recovered, belonging to four orders and 19 families of *Ca*. Saccharimonadia, including previously unreported lineages in activated sludge. These bins had small genomes (approximately 0.46–1.73 Mbp) with limited metabolic capabilities, indicating dependency on other microorganisms. Notably, the order *Ca*. Saccharimonadales retained a type IV secretion system and effector gene cluster for parasitic interactions with the hosts, suggesting that *Ca*. Saccharimonadales bacteria may exhibit a parasitic lifestyle. Co‐occurrence network analysis showed that members of the order *Ca*. Saccharimonadales were significantly correlated with multiple lineages, including *Actinobacteriota*, for which a parasitic relationship has been previously demonstrated. Our results shed light on the potential ecophysiology of the diverse members of *Ca*. Saccharimonadia, providing a comprehensive understanding of *Ca*. Saccharimonadia in activated sludge.

## Graphical abstract

*Candidatus* Saccharimonadia is ubiquitously found in activated sludge processes, but its ecophysiological characteristics remain poorly understood. Metagenome analysis of *Ca*. Saccharimonadia in size‐fractionated activated sludge revealed that the epiparasitic lifestyle may extend to diverse lineages of *Ca*. Saccharimonadia in activated sludge, including rare and previously uncharacterized members.

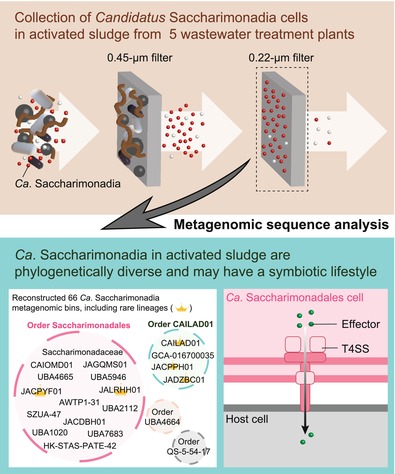

## 1 Introduction

The phylum *Minisyncoccota* (formerly known as candidate phyla radiation and *Candidatus* Patescibacteria) (Nakajima et al. 2025) is a major bacterial phylogenetic group that includes various uncultivated classes, such as *Candidatus* Saccharimonadia, *Candidatus* Gracilibacteria, and *Candidatus* Microgenomatia, and represents > 15% of all bacterial diversity (Brown et al. 2015). For brevity, we hereafter follow the genome taxonomy database (GTDB) taxonomy (Parks et al. 2022) and omit “*Candidatus*” when referring to phylogenetic groups that are not validly published in the phylum *Minisyncoccota*. A common feature of bacteria belonging to *Minisyncoccota* is their small cell size (0.2–0.4 μm) (He et al. 2015; Kuroda et al. 2022; Nakajima et al. 2025) and extremely reduced genome sizes (1.1 ± 0.2 Mbp) (Kagemasa et al. 2022; Tian et al. 2020). In addition, they often lack pathways for the biosynthesis of amino acids, lipids, and nucleotides, suggesting that a predatory or symbiotic lifestyle (e.g., commensal, mutualistic, or parasitic relationships) is widespread within this phylum (Brown et al. 2015; Castelle and Banfield 2018; Castelle et al. 2018; Nakai 2020). Several *Minisyncoccota* bacteria have been observed to have symbiotic relationships with other microorganisms: Saccharimonadia with *Actinobacteriota* (formerly *Actinobacteria*) (Batinovic et al. 2021; He et al. 2015), Gracilibacteria (formerly GN02) with Gammaproteobacteria (Moreira et al. 2021; Yakimov et al. 2022), class‐level uncultured clade JAEDAM01 (formerly BD1‐5) with *Betaproteobacteria* (Fujii et al. 2024), and *Minisyncoccia* with methanogenic archaea (Kuroda et al. 2024; Nakajima et al. 2025).

*Minisyncoccota* are present in both, natural (Castelle et al. 2018; He et al. 2021) and artificial (Hu et al. 2023; Wang, Zhang, et al. 2023) ecosystems. Activated sludge treating sewage in wastewater treatment plants (WWTPs) is a stable habitat (Wang, Gallagher, et al. 2023). The extensive distribution of Saccharimonadia in the activated sludge of WWTPs in different regions and countries (Fujii et al. 2022; Kagemasa et al. 2022; Singleton et al. 2021; Xia et al. 2018; Zhang et al. 2020) and their crucial roles in forming and stabilising floc, bulking, and foaming (Batinovic et al. 2021; Kindaichi et al. 2016; Nielsen et al. 2009) have been reported. Despite the significant impact of Saccharimonadia on WWTP operations and wastewater treatment water quality, its phylogenetic diversity and metabolic potential in activated sludge remain poorly understood.

Recently, a new approach was proposed to efficiently collect genetic information on small cell‐sized microorganisms in activated sludge (Kagemasa et al. 2022). This approach is based on the filtration of activated sludge into the 0.45–0.22 μm fraction. The size‐fractionation approach enriches Saccharimonadia bacterial cells from the activated sludge microbiome and facilitates the recovery of their genomic information. In the present study, we aimed to investigate the metabolic potential of Saccharimonadia in activated sludge systems. To predict the lifestyles of phylogenetically diverse members of Saccharimonadia, we applied the size‐fractionation approach to activated sludge from five WWTPs at different locations with different treatment methods. Size‐fractionated activated sludge samples were subjected to shotgun metagenomic sequencing for metabolic reconstruction. In addition, we included an earlier metagenomic dataset of size‐fractionated, conventional activated sludge (CAS) sample (Kagemasa et al. 2022) for comparative genomic analysis. Furthermore, we predicted the potential hosts of the order Saccharimonadales via 16S rRNA gene‐based co‐occurrence network analysis using 99 activated sludge samples from a WWTP.

## 2 Materials and Methods

### 2.1 Sample Collection and Size‐Fractionation

Activated sludge samples were obtained from five WWTPs with three treatment methods (four CAS processes: MGA, HAC, BEP, and NRA; one anaerobic‐anoxic‐oxic [A2O] process named NRA_A2O; and one nitrification and denitrification process named YHG; Table S1). Activated sludge from the MGA WWTP was sampled weekly from October 2018 to November 2020, whereas the others were sampled once between September and November 2021 (Table S1). In the NRA WWTP, the A2O and CAS processes were employed. Wastewater from the primary sedimentation tank was separated into two treatment trains for the A2O and CAS processes.

Size‐fractionation of the activated sludges was performed according to the method described by Kagemasa et al. (2022). Briefly, activated sludge samples were centrifuged (7000 × *g* at 4°C for 5 min). The supernatant was subjected to size‐fractionation using first, a 0.45‐μm filter (a Stericup filter unit [filter area: 40 cm^2^, PVDF membrane, S2HVU02RE, Merck KGaA, Darmstadt, Germany]), followed by a 0.22‐μm filter (PVDF membrane, S2GVU02RE). Samples were stored at −20°C until further use.

### 2.2 DNA Extraction

DNA was extracted from the unfractionated sludge sample using the ISOIL for Beads Beating Kit (NIPPON GENE Co. Ltd., Tokyo, Japan). For the fractionated samples, half of the filter (approximately 20 cm^2^) was subjected to DNA extraction. The filter was finely chopped into small pieces, immersed in 1.055 mL of Proteinase K solution (0.1 mg/mL in 50 mM Tris–HCl [pH 8.0], 100 mM CaCl_2_, and 0.5% SDS), and incubated for 3 h at 37°C. After incubation, 600 μL of the solution was purified using the ISOIL for Beads Beating kit (DNA purification process only).

### 2.3 Sequencing and Primary Metagenome Analysis

Library preparation and sequencing of the fractionated samples were performed by Takara Bio Inc. (Shiga, Japan). In brief, library preparation was performed using the ThruPLEX DNA‐seq Kit (Takara Bio Inc.) according to the ThruPLEX DNA‐seq Kit User Manual. The DNA was physically fragmented to a few hundred bp using the Acoustic Solubilizer Covaris (Covaris Inc.) and size‐sorted by Agencourt AMPure XP (Beckman Coulter Inc., Brea, CA, USA). A DNA Unique Dual Index Kit 96 U (Takara Bio Inc.) was used for indexing. Sequencing was performed using a NovaSeq6000 (Illumina Inc., San Diego, CA, USA) with the NovaSeq6000 S4 Reagent Kit v1.5 (Illumina Inc.).

Sequencing data were analysed as previously described (Kagemasa et al. 2022). Sequence data were initially subjected to a quality check using Trimmomatic ver. 0.39 (SLIDINGWINDOW:6:30 MINLEN:100) (Bolger et al. 2014). Each sample was assembled individually using MEGAHIT ver. 1.2.9 (k‐min 27, k‐max 141, k‐step 12) (Li et al. 2015, 2016), and contigs below 2500 bp were removed. The contigs were subsequently binned using the MetaBat2 ver. 2.15 (default settings) (Kang et al. 2019), MaxBin 2 ver. 2.2.7 (markerset 40) (Wu et al. 2014), Vamb ver. 3.0.2 (default settings) (Nissen et al. 2021), and MyCC (MyCC_2017.ova) (Lin and Liao 2016). All four sets of binned metagenomes were merged into one set using DAS Tool ver. 1.1.2 (default settings) (Sieber et al. 2018). The optimised non‐redundant bins were dereplicated using dRep ver. 3.2.0 (Olm et al. 2017) with the following parameters: ‐comp, 50; ‐con, 10. Quality checks of the bins were performed using a two‐step approach with CheckM ver. 1.0.7 (Park et al. 2020) and CheckM2 ver. 1.1.0 (Chklovski et al. 2023). Initially, bins were screened with CheckM using the cpr_43_markers.hmm marker set for *Minisyncoccota* genomes. Bins that passed this initial screening were then subjected to a selection of the final set of bins using CheckM2. A consistent quality threshold of completeness greater than or equal to 50% and less than 10% contamination was maintained throughout the process. The selected bins were phylogenetically classified using the Genome Taxonomy Database Toolkit (GTDB‐Tk) ver. 2.3.2 (r214) (Chaumeil et al. 2022), and those classified as Saccharimonadia were selected for further analysis. Nucleotide sequence data are available in the DDBJ Sequence Read Archive accession number PRJDB20466.

### 2.4 Selection of Reference Genomes

This study included the genomes of Saccharimonadia recovered from a variety of samples worldwide, including activated sludge samples, as reference genome sequences for subsequent analysis (Table S2). Reference genomes were also quality‐checked using the two‐step approach with CheckM ver. 1.0.7 and CheckM2 ver. 1.1.0, and only those passing the aforementioned quality threshold were selected. For quality‐filled genomes, phylogenetic analysis was performed using the GTDB‐Tk ver. 2.3.2 (r214). The 67 reference genomes of Saccharimonadia were selected (Table S2), from sludge samples (activated sludge: *n* = 44, primary sludge: *n* = 1, digested sludge: *n* = 1) and wastewater samples (*n* = 16) from WWTPs worldwide (Japan (Fujii et al. 2022), China (Wang et al. 2021), Germany (Schneider et al. 2021), Denmark (Albertsen et al. 2013; Singleton et al. 2021), and Australia (Batinovic et al. 2021)), mammalian oral samples (*n* = 3) (He et al. 2015; McLean et al. 2020), and aquifer sediment samples (*n* = 2) (Brown et al. 2015; Kantor et al. 2013). The following reference sequences used are complete genomes: *Ca*. Saccharimonas aalborgensis (Albertsen et al. 2013) and *Ca*. Mycosynbacter amalyticus (Batinovic et al. 2021) recovered from activated sludge samples, *Ca*. Nanosynbacter lyticus strain TM7x (He et al. 2015) recovered from an oral sample, and RACC3_TM7_1 (Kantor et al. 2013) and GWC2_44_17 (Brown et al. 2015) recovered from aquifer sediment samples.

### 2.5 Construction of a Genome Tree

A genome tree was constructed using the bins obtained (*n* = 65 [including seven bins recovered from the fractionated samples of MGA WWTPs]; Table S3), reference genomes (Table S2), and saccharimonadial sequences from GTDB‐Tk ver. 2.3.2 (r202). The sequences were aligned using GTDB‐Tk ver. 2.3.2 (r214), and the aligned sequences were used to construct a genome tree using the IQ‐Tree 2 ver. 2.1.2‐bet (Minh et al. 2020) using the Maximum Likelihood method (IQ‐TREE multicore, <alignmentfile>bb 1000 −m LG + G4 + FO + I) (He et al. 2021).

### 2.6 Genome Size and the Number of Coding Sequence Estimation

The genome size of Saccharimonadia bins recovered from fractionated samples (*n* = 66, Table S3) was compared to that of Saccharimonadia present in mammalian and insect bodies (intestine and stomach), the representative Saccharimonadia (*Ca*. Nanosynbacter lyticus strain TM7x (He et al. 2015), *Ca*. Saccharimonas aalborgensis (Albertsen et al. 2013) and *Ca*. Mycosynbacter amalyticus (Batinovic et al. 2021)), and representative free‐living ultramicrobacteria. Representative free‐living ultramicrobacteria include *Aurantimicrobium minutum* KNC^T^ (Nakai et al. 2015), *Ca*. Fonsibacter ubiquis LSUCC0530 (Henson et al. 2018), *Ca*. Pelagibacter ubique SAR11 HTCC1062 (Rappé et al. 2002), *Polynucleobacter necessarius* subsp. *asymbioticus* QLW‐P1DMWA‐1^T^ (Meincke et al. 2012), and *Rhodoluna lacicola* MWH‐Ta8^T^ (Hahn et al. 2014), all of which have streamlined genomes. Genome size and coding sequence (CDS) of Saccharimonadia in mammalian and insect bodies (intestine, stomach) and representative free‐living ultramicrobacteria were obtained from the Integrated Microbial Genomes & Microbiomes system (IMG: https://img.jgi.doe.gov/m/) (Chen et al. 2021). These data were obtained using the Quick Genome Search function in IMG (keywords: for example, “*Candidatus* Saccharibacteria [narrowing the conditions with the isolation source with mammalian or insect body],” and “*Aurantimicrobium minutum*”). Genome size and CDS of the bins obtained from the fractionated samples, and representative Saccharimonadia genomes were determined using CheckM2 ver. 1.1.0 and Prokka ver. 1.14.6 (Seemann 2014).

### 2.7 Functional Annotation of Genes

The bins obtained (*n* = 65; Table S3) and reference genomes (*n* = 67; Table S2) were annotated using Prokka ver. 1.14.6 and DRAM (use_uniref option with default settings) (Shaffer et al. 2020). For bins belonging to order CAILAD01, additional annotation of ATPase genes was performed using Blast KEGG Orthology And Links Annotation (BlastKOALA) (Kanehisa et al. 2016).

We inferred whether the bins recovered from the fractionated samples and the reference genomes possessed genes for pili, type IV secretion system (T4SS), and effector cluster by performing a BLASTp‐based homology search. An e‐value cut‐off of 1e‐5 was chosen to ensure a stringent homology search. BLASTp‐based homology search was performed against the amino acid sequences of the complete genomes of Saccharimonadia pili and *Ca*. Nanosynbacter lyticus strain TM7x as a T4SS and effectors (Hendrickson et al. 2022; McLean et al. 2020).

### 2.8 Amplicon Sequence Analysis of the 16S rRNA Gene

Amplicon sequencing was performed to target the V3–V4 region of the 16S rRNA gene (Ni et al. 2020) using unfractionated samples (*n* = 99). Primers without overhang sequences were used for the initial polymerase chain reaction (PCR) to minimise PCR bias (Berry et al. 2011). Primer sets of 341F (5′‐CCT AYG GGR BGC ASC AG‐3′) and 806R‐mix (a mixture of 806R [5′‐GGA CTA CHV GGG THT CTA AT‐3′] and 806R‐P [5′‐GGA CTA CCA GGG TAT CTA AG‐3′] at a ratio of 30:1) were used (Matsubayashi et al. 2017). Sequencing was performed using an Illumina MiSeq sequencer (Illumina Inc.) with the MiSeq Reagent Kit v3 600‐cycles (Illumina Inc.) following the manufacturer's instructions.

Raw 16S rRNA gene sequences were analysed using QIIME 2 ver. 2024.10 (Bolger et al. 2014), according to a previous study (Kuroda et al. 2022). Briefly, demultiplexed sequences from each sample were quality‐filtered, trimmed, denoised, and merged, and the chimeric sequences were identified and removed using the QIIME2 dada2 plugin to obtain a feature table of amplicon sequence variants (Callahan et al. 2016). The 16S rRNA gene sequences were clustered by ≥ 97% similarity to operational taxonomic units (OTUs) using VSEARCH software (Rognes et al. 2016). The QIIME2 feature‐classifier classify‐sklearn plugin (Abraham et al. 2014) was used to align the amplicon sequence variant sequences with a pre‐trained GTDB SSU database release 214 (trained to the ssu_all_r214.fa classifier using RESCRIPt) to generate a taxonomy table (Parks et al. 2022; Robeson et al. 2021).

### 2.9 Calculation and Mapping of Correlation Coefficients Between OTUs

To elucidate the relationship between Saccharimonadales and other co‐existing bacteria, OTUs, a proxy for species, were extracted based on a relative abundance of ≥ 0.1%. The Spearman's rank correlation coefficient was calculated for each OTU using Past 5 software (Hammer et al. 2001). The co‐occurrence relationships among OTUs belonging to Saccharimonadia and *Actinobacteriota*, defined as an absolute value of Spearman's rs greater than or equal to 0.4 and a *p*‐value of less than 0.05, were graphically represented using Cytoscape ver. 3.10.3 (Shannon et al. 2003).

## 3 Results

### 3.1 Saccharimonadia Metagenomic Bins Recovered From Size‐Fractionated Activated Sludge

**FIGURE 1:**
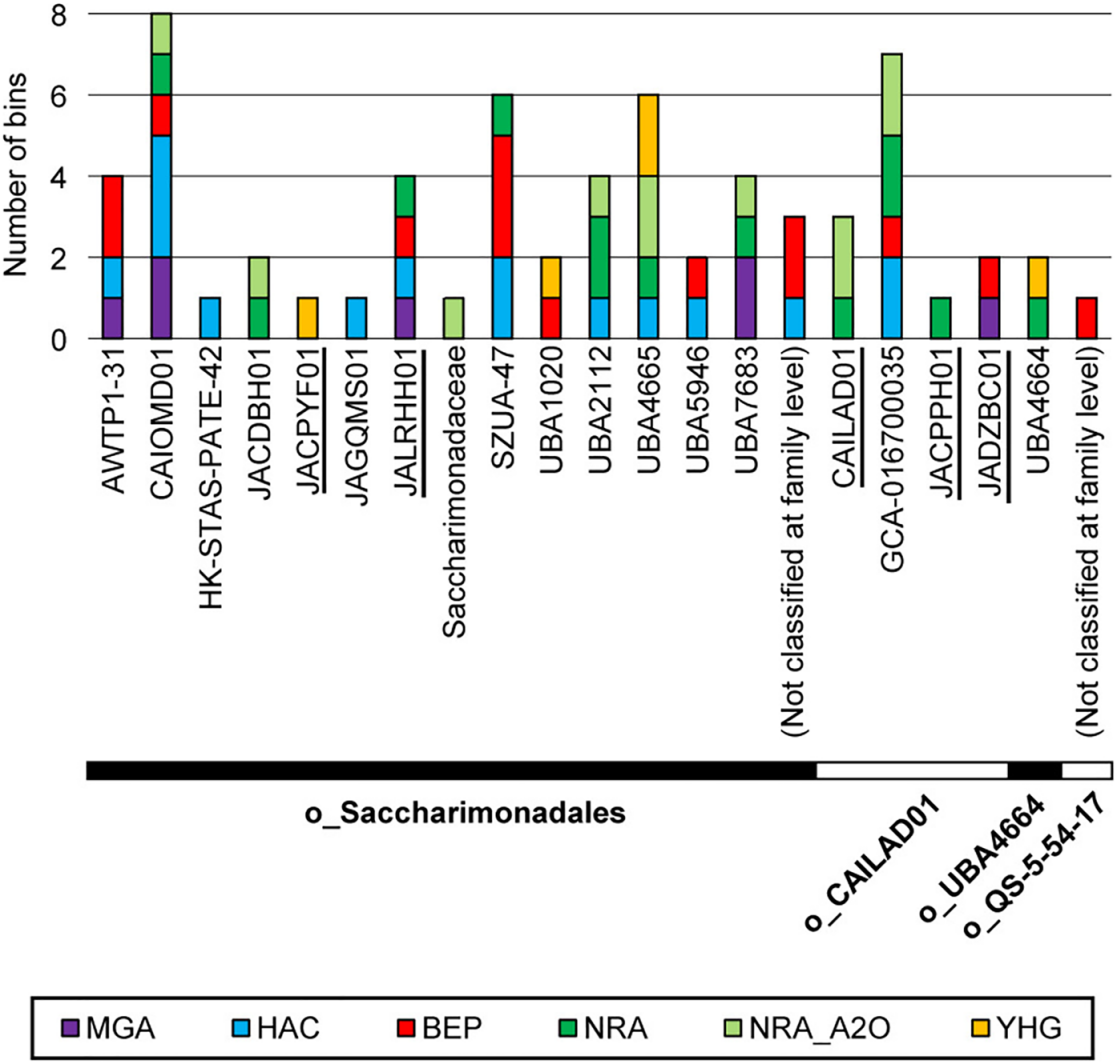
Number of *Candidatus* Saccharimonadia bins reconstructed from the fractionated activated sludge samples. Each lineage name indicates the family‐level taxonomy, while those with “o_” in front of the name of the lineage indicate the order‐level taxonomy. The family name with an underscore indicates that the bins in these lineages have not been recovered from activated sludge previously (Albertsen et al. 2013; Batinovic et al. 2021; Fujii et al. 2022; Schneider et al. 2021; Singleton et al. 2021; Wang et al. 2021) (Table S3).

We reconstructed 58 metagenomic bins of Saccharimonadia with qualities of ≥ 70% completeness and < 10% contamination from the five size‐fractionated activated sludge samples (Table S3). The numbers of reconstructed bins for Saccharimonadia were 15 in the HAC sample, 14 in the BEP sample, 13 in the NRA_A2O sample, 11 in the NRA sample, and 5 in the YHG sample (Tables S3 and S4). Seven bins of Saccharimonadia were also recovered from the MGA sample in our previous study (Kagemasa et al. 2022); thus, 65 bins were subjected to further analysis. These bins belonged to four orders (Figure 1): Saccharimonadales (49 bins), CAILAD01 (13 bins), UBA4664 (two bins), and QS‐5‐54‐17 (one bin).

The reconstructed saccharimonadial bins belonged to 19 families and 21 genera based on the GTDB classification (Tables S3 and S4), some of which were rarely found in unfractionated activated sludge samples (Figure 1 and Figure S1). Bins belonging to family‐level uncultured lineages, that had not been previously found in unfractionated activated sludge (Albertsen et al. 2013; Batinovic et al. 2021; Fujii et al. 2022; Schneider et al. 2021; Singleton et al. 2021; Wang et al. 2021), were recovered from the fractionated samples; that is, JALRHH01 and JACPYF01 in the order Saccharimonadales and CAILAD01, JACPPH01, and JADZBC01 in the order‐level uncultured lineage CAILAD01.

### 3.2 Genome Size and Potential Metabolic Characteristics of Saccharimonadia Bins Recovered From the Fractionated Samples

**FIGURE 2:**
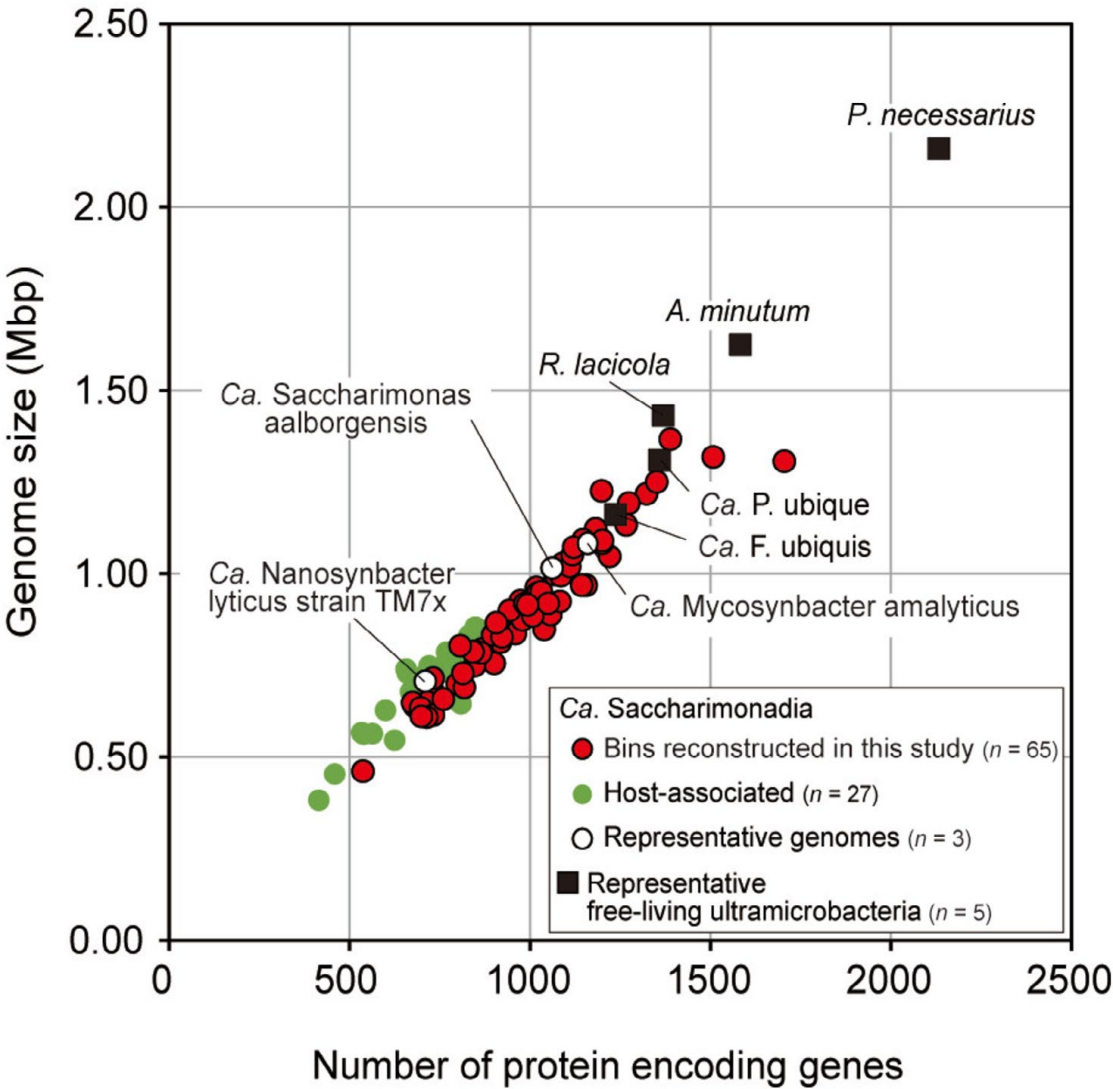
Predicted genome size and number of protein‐encoding genes for *Candidatus* Saccharimonadia. This figure shows the genome size and number of protein‐encoding genes of *Ca*. Saccharimonadia (circles) based on bins reconstructed in this study (red circles) and publicly available genomes (Integrated Microbial Genomes & Microbiomes system [IMG]). Host‐associated *Ca*. Saccharimonadia bacteria in mammalian and insect bodies (intestine and stomach) are indicated by green circles, and representative *Ca*. Saccharimonadia genomes are indicated by white circles. Genomes of known, free‐living ultramicrobacteria with genome streamlining are also marked (black squares): *Aurantimicrobium minutum*, *Ca*. Fonsibacter ubiquis, *Ca*. Pelagibacter ubique, *Polynucleobacter necessarius* subsp. *asymbioticus*, and *Rhodoluna lacicola*.

The average genome size of the 65 metagenomic bins of Saccharimonadia was 0.91 Mbp (minimum–maximum: 0.46–1.73 Mbp, Table S3)—smaller than that of free‐living ultramicrobacteria (average: 1.33 Mbp, minimum–maximum: 1.12–1.44 Mbp, *n* = 13, Figure 2). This result is also consistent with the average genome size of 0.89 Mbp (0.53–1.26 Mbp, *n* = 44, Table S2) for Saccharimonadia found in activated sludge (Albertsen et al. 2013; Batinovic et al. 2021; Fujii et al. 2022; Schneider et al. 2021; Singleton et al. 2021; Wang et al. 2021), including the complete genomes of *Ca*. Saccharimonas aalborgensis (genome size: 1.01 Mbp) and *Ca*. Mycosynbacter amalyticus (1.08 Mbp).

**FIGURE 3:**
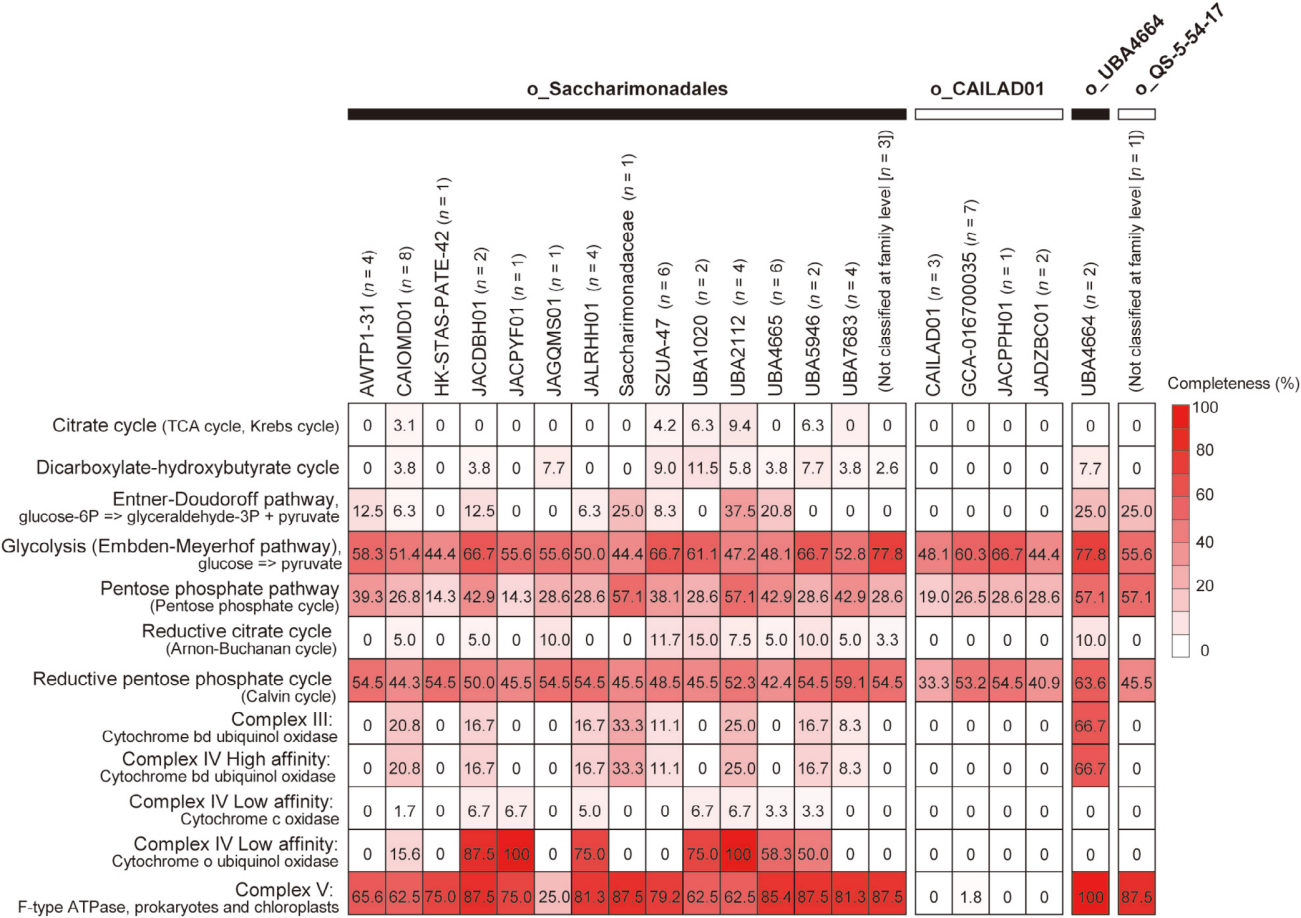
Completeness of metabolic pathways of *Candidatus* Saccharimonadia bins reconstructed from the fractionated activated sludge samples. “o_” indicates an order name; without “o_” indicates a family name. Numbers in parentheses after the phylogenetic name indicate the number of bins reconstructed from the fractionated sample.

**FIGURE 4:**
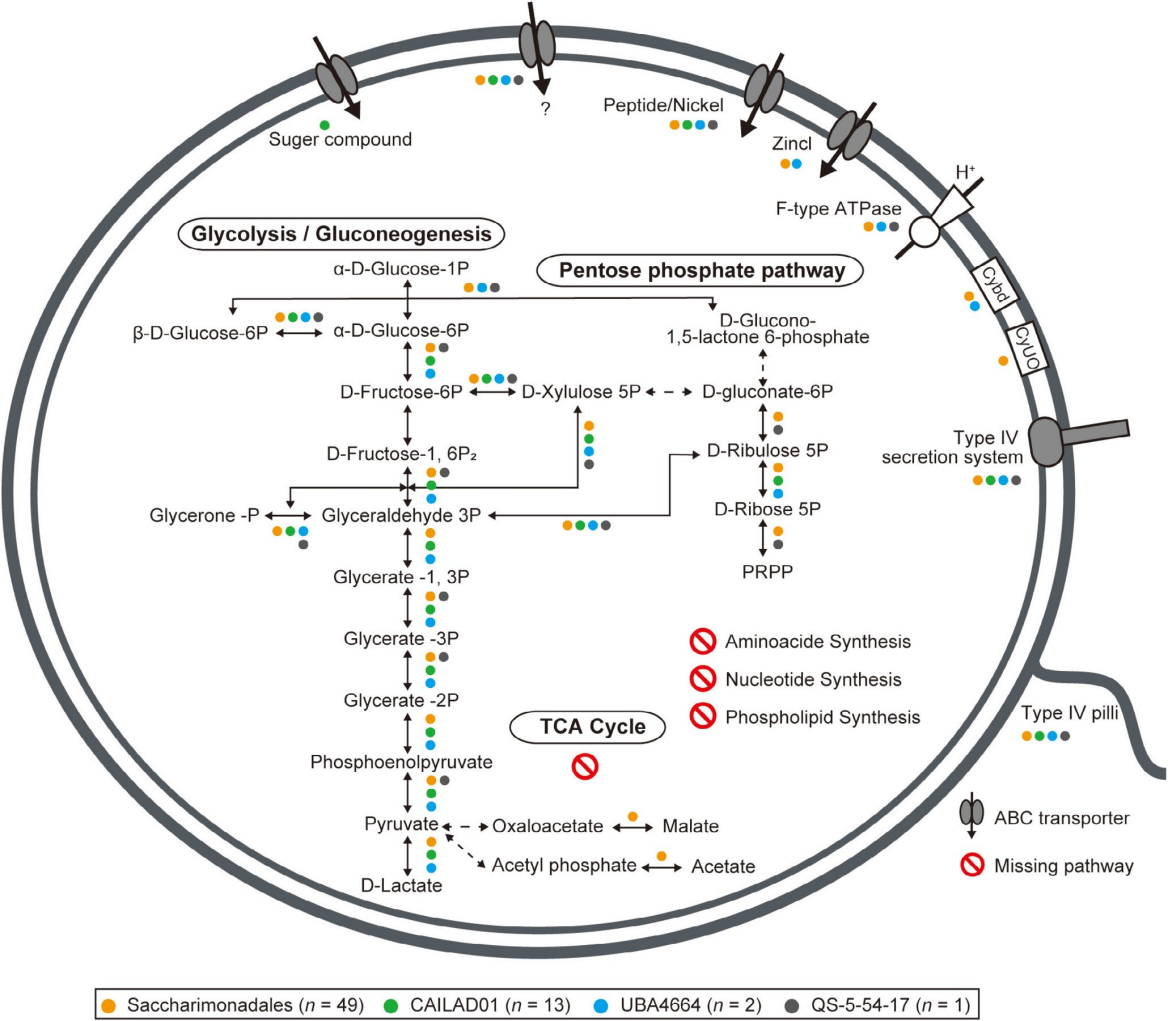
Metabolic potential of *Candidatus* Saccharimonadia predicted from the bins reconstructed from the fractionated activated sludge samples. Coloured circles indicate that the corresponding gene may be retained in each taxonomy. Cybd and CyUO indicate Cytochrome bd ubiquinol oxidase and Cytochrome o ubiquinol oxidase, respectively.

The predicted metabolic potential of Saccharimonadia based on metagenomic analysis in this study is shown in Figures 3 and 4. Almost all bins encoded genes related to the glycolytic pathway (Embden‐Meyerhof‐Parnas pathway, 65 bins) and the pentose phosphate pathway (64 bins) (Figure 3). Similar results were obtained using the reference genomes (Figure S2). These bins and reference genomes did not contain complete sets of these pathways; however, most bins (62 bins) and reference genomes (56 genomes) encoded transketolase, a key factor linking these pathways (Figure 4). The bins retained a few genes for the electron transfer chain and the tricarboxylic acid (TCA) cycle (Figures 3 and 4). The biosynthetic pathway downstream of the pentose phosphate pathway was absent. Only bins belonging to the families CAIOMD01, JACDBH01, JACPYF01, JALRHH01, UBA1020, UBA2112, UBA4665 and UBA5946 in the order Saccharimonadales possessed cytochrome o ubiquinol oxidase. Genes that converted pyruvate to lactate (d‐lactate dehydrogenase), acetate (acetate kinase) and malate (malate dehydrogenase) were also identified. Additionally, most bins comprised complete or nearly complete F‐type ATPases (45 bins, Figures 3 and 4), *FtsX* (59 bins) and *FtsE* (57 bins) genes that prevented morphological abnormalities and cell death during cell division (Du et al. 2019; Meier et al. 2017; Pichoff et al. 2019).

### 3.3 Recovery of Genes Related to Parasitic/Symbiotic Interactions

Saccharimonadia isolates have lifestyles of epibionts on the surface of the host, as confirmed by microscopic observation of cultured strains (Batinovic et al. 2021; Cross et al. 2019; He et al. 2015). In a previous study, ATPase and type IV pili (T4P) were predicted to function in the attachment and motility on host surfaces (Seymour et al. 2023). Most bins possessed genes encoding F‐type ATPases (Figure 4, Table S5) and T4P (Table S6). Among the Saccharimonadia bins recovered from the size‐fractionated samples, 62 bins (excluding HAC_MetaBin.171, HAC_MetaBin.123, and NRA_MetaBin.26) retained the T4P operon (Table S6), which includes genes for major pilins (*PilA*), prepilin peptidase (*PilD*), minor pilins (*PilE*), inner membrane core protein (*PilC*), inner membrane accessory protein (*PilM*), and retraction ATPase (Leighton et al. 2015; Melville and Craig 2013; Xie et al. 2022).

**FIGURE 5:**
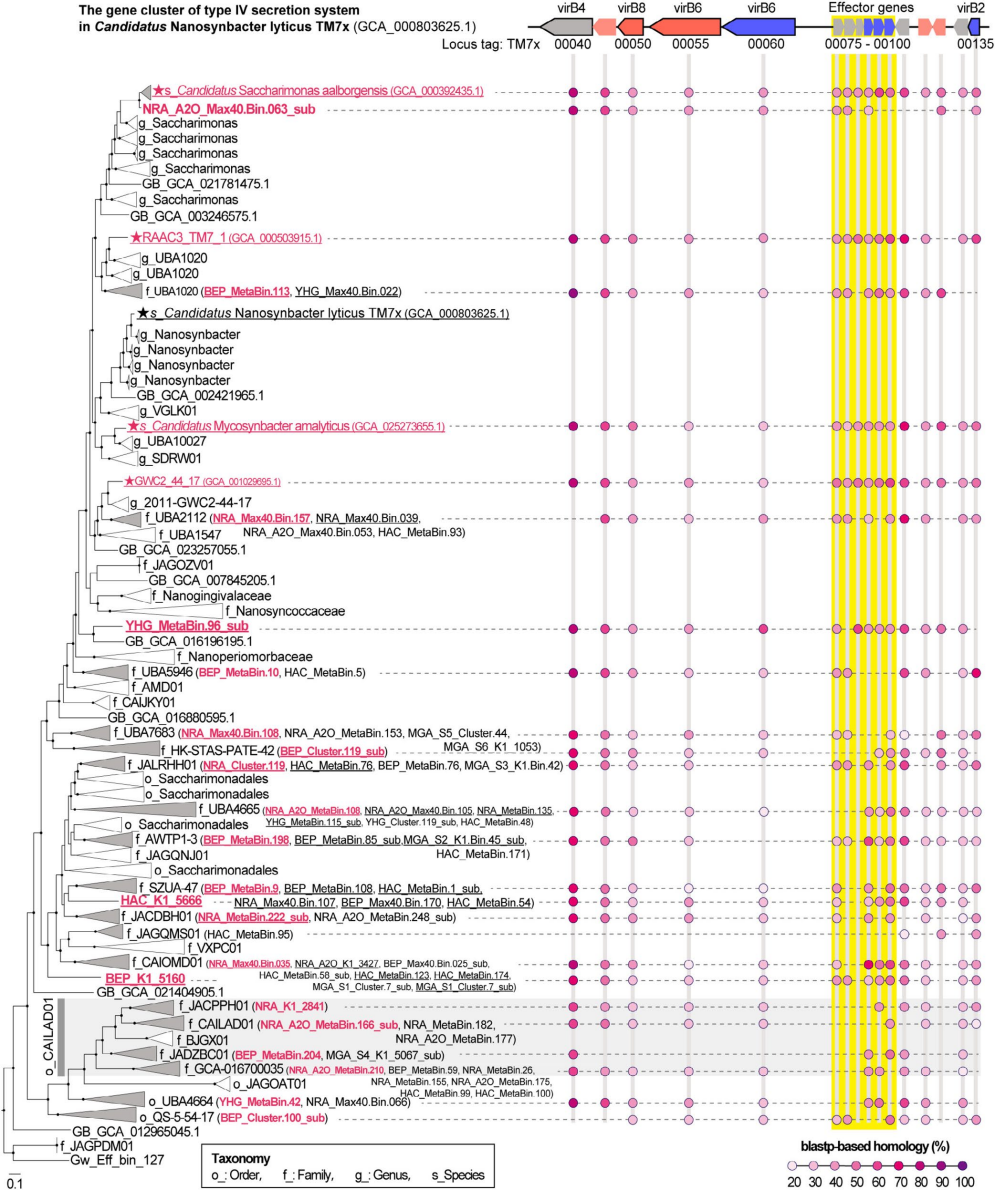
Genome‐based maximum likelihood phylogenetic tree of *Ca*. Saccharimonadia with homology search results for the type IV secretion system (T4SS) gene clusters of *Candidatus* Nanosynbacter lyticus TM7x. Black circles at the nodes indicate bootstrap values of 95% or higher (1000 replicates). Names with a star indicate a complete genome. Bin IDs reconstructed from the fractionated samples are shown in brackets. Clades indicated by the triangle containing the bins obtained in this study are coloured grey. The bins/reference genome IDs are shown in red, indicating the presence or absence of the genes homologous to the genes of T4SS of *Ca*. Nanosynbacter lyticus strain TM7x. Bins/reference genomic IDs that may have retained effector clusters are underlined. Pink coloured circles indicate a BLASTp‐based homology (≤ 1e‐5 e‐value) with the T4SS genes of *Ca*. Nanosynbacter lyticus strain TM7x.

In parasitic interactions, it is clear that effectors that alter host physiology and the secretory system to transport them are important (Kuroda et al. 2022; Moreira et al. 2021). BLASTp‐based homology search with 65 reconstructed bins against known T4SS and effector genes of epiparasitic bacterium *Ca*. Nanosynbacter lyticus strain TM7x showed that many bins encoded these genes in their genomes (Figure 5). The bins retained some *virB* genes (Table S7), that is, *virB2* (found in 23 bins) as a major component of the pilus, *virB4* (found in 47 bins) essential for both the assembly of the system and substrate transfer, and *virB6* (found in 40 bins) and *virB8* (found in 47 bins) for inner‐membrane components with unknown roles but essential for the functions of T4SS (Hendrickson et al. 2022; McLean et al. 2020; Walldén et al. 2012). In addition, 31 bins (belonging to the orders Saccharimonadales and QS‐5‐54‐17) comprised effector gene clusters (Figure 5, Table S7). Similar results were obtained for the reference genomes (Table S8). Of the 43 reference genomes with effector gene clusters, 42 belonged to the order Saccharimonadales and 1 belonged to the order‐level lineage CAILAD01.

### 3.4 Bacteria Significantly Correlated With Saccharimonadales in Activated Sludge

Co‐occurrence analysis was conducted to identify the potential bacterial hosts of Saccharimonadia in the activated sludge. Spearman's rank correlation coefficients (*rs* ≥ 0.4 and *p* < 0.05) were calculated between the saccharimonadial OTUs obtained from amplicon sequencing and other bacterial OTUs with an average relative abundance of more than 0.1% (*n* = 206). All saccharimonadial OTUs with an average relative abundance of 0.1% in the activated sludge belonged to the order Saccharimonadales. The OTUs of *Acidobacteriota* (Spearman's rs: 0.43–0.52, *n* = 2), *Actinobacteriota* (0.43–0.84, *n* = 7), *Bacillota* (0.42–0.76, *n* = 5), Bacillota_A (0.41–0.68, *n* = 11), Bacillota_B (0.43–0.70, *n* = 1), Bacillota_C (0.40–0.51, *n* = 1), *Bacteroidota* (0.40–0.92, *n* = 33), *Chloroflexota* (0.42–0.86, *n* = 11), *Vulcanimicrobiota* (0.73, *n* = 1), *Fusobacteriota* (0.50–0.60, *n* = 2), *Myxococcota* (0.43–0.77, *n* = 2), *Minisyncoccota* (*Ca*. Gracilibacteria, 0.43–0.54, *n* = 1), *Pseudomonadota* (0.40–0.81, *n* = 67), and *Verrucomicrobiota* (0.43–0.50, *n* = 3) showed positive correlations with the OTUs of Saccharimonadales (*n* = 13).

**FIGURE 6:**
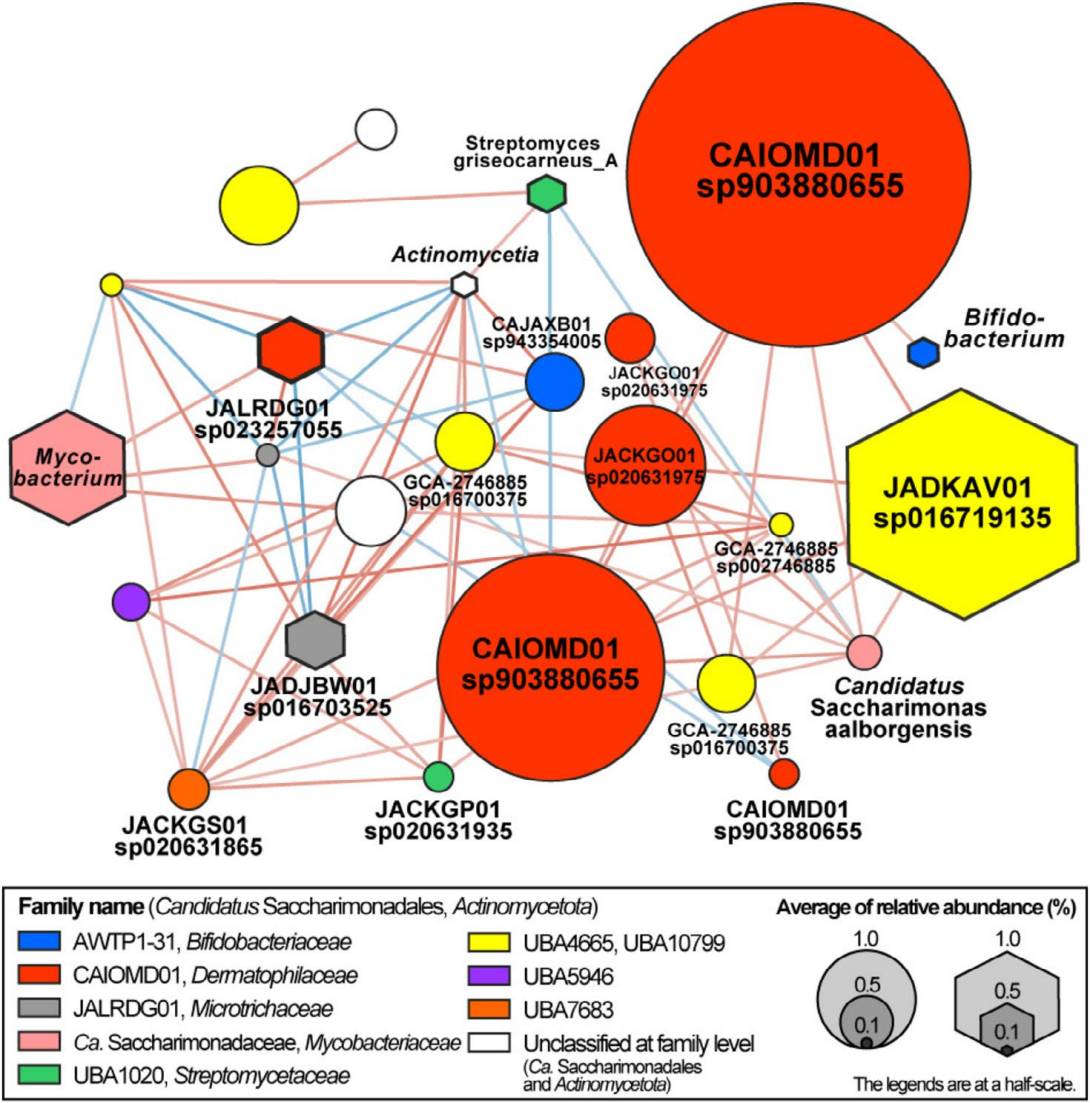
Co‐occurrence analysis of *Candidatus* Saccharimonadales and *Actinobacteriota*. The nodes in our visual representation represent operational taxonomic units (OTUs), with a circle indicating *Ca*. Saccharimonadia and a hexagon indicating OTUs belonging to *Actinobacteriota*. The size of each node is directly proportional to the average relative abundance of OTUs in the MGA_unfractionated sample (*n* = 99). The edge colour is shown in red when the correlation between OTUs is positive and in blue when the correlation is negative. Transparency of edge indicates correlation coefficient between OTUs (nodes), i.e., darker lines indicate higher correlation coefficient values.

Of the bacteria that have shown a positive correlation with Saccharimonadales, *Actinobacteriota* are known to be closely related to them because all known hosts of Saccharimonadales belong to *Actinobacteriota* (Batinovic et al. 2021; Bor et al. 2020; Chipashvili et al. 2021; He et al. 2015; Utter et al. 2020; Xie et al. 2021). The co‐occurrence relationship between Saccharimonadales and *Actinobacteriota* is shown in Figure 6. In the present study, *Actinobacteriota* OTUs belonging to different genera and species were found to be correlated with several Saccharimonadales OTUs, highlighting the potential relationship between Saccharimonadales and *Actinobacteriota* spp. One Saccharimonadales OTU, for instance, was found to be positively correlated with six *Actinobacteriota* OTUs (Figure 6).

## 4 Discussion

### 4.1 Diversity of Saccharimonadia in Activated Sludge

In this study, we successfully recovered the phylogenetically diverse saccharimonadial bins (four orders, but mainly the order Saccharimonadales [Figure 1]) from the 0.45–0.22 μm‐fraction of activated sludge samples from WWTPs in Japan. Many Saccharimonadia families have previously been detected in activated sludge (Albertsen et al. 2013; Batinovic et al. 2021; Fujii et al. 2022; Schneider et al. 2021; Singleton et al. 2021; Wang et al. 2021), suggesting their wide distribution in activated sludge (Figure 1). In contrast, bins belonging to several families (JALRHH01 and JACPYF01 belonging to the order Saccharimonadales, and CAILAD01, JACPPH01, and JADZBC01 belonging to the order‐level uncultured lineage CAILAD01; Figure 1) have only been found in natural environments, such as estuarine water, groundwater, and soil (He et al. 2021; Mosley et al. 2022; Ortiz et al. 2021; Zhou et al. 2022). In addition, 41 bins showed an average nucleotide identity [ANI] value less than 95% of the reference genome sequences in the GTDB r214 (Chaumeil et al. 2022). The ANI value below 95% is an established threshold for species identification (Richter and Rosselló‐Móra 2009). Therefore, it is suggested that activated sludge may present a higher diversity of Saccharimonadia species than previously expected (Fujii et al. 2022; Schneider et al. 2021; Singleton et al. 2021).

### 4.2 Limited Metabolic Capacity of Saccharimonadia

Saccharimonadia has a highly reduced genome with a limited metabolic capacity (Castelle et al. 2018). Our metagenomic analysis also suggested a limited metabolic capacity of Saccharimonadia in activated sludge, i.e., unable to synthesise nucleotides, amino acids, or phospholipid synthesis *de novo* (Figure 4). Although Saccharimonadia lacked complete pathways for carbohydrate metabolism, it possessed part of the glycolytic and pentose phosphate pathways (Figures 3 and 4). The combination of these two metabolic pathways potentially degraded glucose to pyruvate. The saccharimonadial bins possessed genes for converting pyruvate to lactate, acetate, and malate, and the lack of a TCA cycle supported fermentative metabolism (Figure 4). These results are consistent with those from previous studies (Albertsen et al. 2013; Baker 2021; Castelle and Banfield 2018; Fujii et al. 2022; Kantor et al. 2013; Wang, Zhang, et al. 2023).

Some specific families of the order Saccharimonadales possessed cytochrome o ubiquinol oxidase (Figures 3 and 4), which is related to the oxygen scavenging system. Cytochrome o ubiquinol oxidase catalyses the reduction of O_2_ to H_2_O and creates a transmembrane proton gradient that provides energy for ATP synthesis (He et al. 2024; Kantor et al. 2013; Lemos et al. 2019; Nelson and Stegen 2015; Starr et al. 2018). The simultaneous presence of cytochrome o ubiquinol oxidase and ATPases (Figures 3 and 4) in the families CAIOMD01, JACDBH01, JACPYF01, JALRHH01, UBA1020, UBA2112, UBA4665 and UBA5946 suggests these families may respire aerobically.

### 4.3 Parasitology of Saccharimonadales Bacteria in Activated Sludge

The average genome size of order Saccharimonadales bacteria, the major group of Saccharimonadia found in activated sludge (Albertsen et al. 2013; Batinovic et al. 2021; Fujii et al. 2022; Schneider et al. 2021; Singleton et al. 2021; Wang et al. 2021), is less than 1 Mbp (Tables S3 and S4). This reduced genome size falls below the 1 Mbp threshold for a bacterial lineage that is obligately associated with the host cytoplasm (McCutcheon and Moran 2010), suggesting that Saccharimonadales in activated sludge are parasitic/symbiotic bacteria. In addition, to compensate for their metabolic deficiencies, these bacteria have retained genes crucial for establishing parasitic/symbiotic relationships (i.e., T4P [Table S6], T4SS, and effectors [Tables S7 and S8]), highlighting their dependence on others (Batinovic et al. 2021; Hendrickson et al. 2022; McLean et al. 2020; Rose et al. 2025). We found that Saccharimonadales in activated sludge have functions necessary for attaching to (Figure 4 and Table S6) and infecting (Figure 5, Tables S7 and S8) hosts. These functions include effector clusters and the T4SS for parasitic interactions. The genes of potential effector clusters (Tables S7 and S8) were homologous to those reported to show increased expression levels during stable symbiosis (Hendrickson et al. 2024, 2022), suggesting that Saccharimonadales use these effector genes to infect the hosts. Based on these findings, Saccharimonadales in the activated sludge are likely epiparasitic bacteria that use the T4P to attach to the host's surface and the T4SS to deliver effector molecules.

The present study found a potential infection‐host relationship between Saccharimonadales and several *Actinobacteriota* bacterial species in activated sludge (Figure 6). In addition to the integrity of mycolic acids, which are key components of the outer cellular envelope of *Actinobacteriota* (Rose et al. 2025), the diversity of effector repertoires (Figure S3, Supporting Information S2) may define Saccharimonadales in the activated sludge, extending to a wide range of *Actinobacteriota* lineages. Effectors in Saccharimonadales not only contribute to establishing a stable symbiotic relationship with the hosts but may also be a potential indicator of a range of hosts (Lee et al. 2020). The number of effector genes and their phylogenetic diversity may reflect the breadth of the host range, given that effectors are mainly encoded through horizontal gene transfer from the hosts (Mak and Thurston 2021). In practice, *Legionella pneumophila* , which hosts a broad range of eukaryotes, acquires and maintains a large effector repertoire and uses different effectors depending on the host (Lee et al. 2020). The effectors encoded by the Saccharimonadales bins/reference genomes were widely distributed within the phylogenetic tree (Figure S3), suggesting that Saccharimonadales may retain a phylogenetically diverse effector repertoire. In addition, Saccharimonadales bacteria in the activated sludge retained a larger number of effector genes than those in the human oral cavity, even though they belonged to the same genus (genus Saccharimonas, Tables S7 and S8), suggesting that they have a wider host range. This is supported by the fact that oral cavity‐inhabiting *Ca*. Nanosynbacter lyticus strain TM7x is hosted by a limited species belonging to the genus *Actinomyces* (Utter et al. 2020), whereas activated sludge‐inhabiting *Ca*. Mycosynbacter amalyticus is hosted by 27 species belonging to various genera of Mycolata groups (Batinovic et al. 2021). This study's co‐occurrence analysis also suggested that Saccharimonadales bacteria in activated sludge have a wide host range. Approximately 50% of the 13 Saccharimonadales OTUs that were positively correlated with *Actinobacteriota* OTUs also showed positive correlations with several *Actinobacteriota* OTUs for one Saccharimonadales OTU (Figure 6). This suggests that Saccharimonadales may have multiple hosts. Therefore, Saccharimonadales bacteria in activated sludge may maintain their populations through parasitism on a wide range of *Actinobacteriota* lineages.

## 5 Conclusions

We revealed the high phylogenetic diversity of Saccharimonadia in activated sludge and predicted their metabolic potential based on saccharimonadial bins obtained by applying a size‐fractionation approach to various activated sludge samples. The reconstructed saccharimonadial bins included rare lineages that have not been previously obtained from activated sludge samples. Comparative genomic analysis revealed that the recovered metagenomic bins lacked several pathways, including *de novo* nucleotide, amino acid, and phospholipid synthesis, as well as a complete TCA cycle, suggesting a parasitic/symbiotic lifestyle. In particular, the major phylogenetic group in activated sludge, the order Saccharimonadales, potentially retained diverse effector genes involved in host parasitism, implying that the interaction between Saccharimonadales bacteria and their hosts was parasitic. Based on their relative abundance, Saccharimonadales bacteria also showed positive correlations with multiple phylotypes of *Actinobacteriota*, with which a parasitic relationship has already been reported. This study revealed that the parasitic lifestyle extended to diverse lineages of Saccharimonadales bacteria in activated sludge. Furthermore, the genomic information on diverse Saccharimonadales members accumulated using size‐fractionation is helpful for determining culture conditions. Further studies on the cultivation of activated sludge Saccharimonadia and evaluation of enzymes associated with host attachment are necessary to elucidate the lifestyles of Saccharimonadales bacteria that inhabit activated sludge processes.

## Author Contributions

**Shuka Kagemasa:** investigation, funding acquisition, writing – original draft, formal analysis, visualisation. **Kyohei Kuroda:** conceptualization, methodology, software, supervision, writing – review and editing, investigation. **Ryosuke Nakai:** investigation, writing – review and editing, funding acquisition, supervision. **Mikiko Sato:** investigation. **Yu‐You Li:** writing – review and editing, resources. **Kengo Kubota:** writing – review and editing, resources, supervision, project administration, funding acquisition.

## Conflicts of Interest

The authors declare no conflicts of interest.

## Supporting information


**Figure S1:** A genome‐based maximum likelihood phylogenetic tree of Candidatus Saccharimonadia. The reconstructed bins in this study are shown in bold and meticulously colour‐coded by the samples: purple for MGA, light blue for HAC, red for BEP, green for NRA, yellowish green for NRA_A2O, and yellow for YHG. A circle next to the bin's ID indicates the average nucleotide identity (ANI) value between the bins and the reference genomes in the genome taxonomy database r214: a black circle for an ANI value between 75 and less than 85, a grey circle for an ANI value between 85 and less than 95, and a white circle for an ANI value greater than 95. No circles are shown, indicating a bin for which it could not be calculated ANI values. Complete genomes are marked with a star. The names of the families for which genomes have not been recovered from activated sludge to date are shown in red. The isolation source and its location are shown in parentheses. Black circles at the nodes indicate bootstrap values of 95% or higher (1000 replicates)
**Figure S2:** Completeness of the metabolic pathways of Candidatus Saccharimonadia reference genomes. “o_” indicates an order name; without “o_,” indicates a family name. Numbers in parentheses after the phylogenetic name indicates the number of reference genomes
**Figure S3:** Phylogenetic tree based on genes homologous to effector genes of Candidatus Nanosynbacter lyticus strain TM7x. After the bin/reference genome names, the underscores indicate the effector gene locus tags. The letters' colours indicate that the red represents the Ca. Nanosynbacter lyticus TM7x strain, the blue represents the bin/reference genome of the order CAILAD01, and the grey represents the outgroup. Complete genomes are marked with a star. The numbers in parentheses (1–6) correspond to the effector gene locus tags of Ca. Nanosynbacter lyticus strain TM7x, TM7x_00090–TM7x_00100, indicating effector genes with homology (≤ 1e‐5 e‐value) to the genes in the bins/reference genomes. The names of the lineages to which the bins/reference genomes to which the genes were detected belong are also shown in parentheses. Black circles at the nodes indicate bootstrap values of 95% or higher (1000 replicates).

**Table S1:** Activated sludge samples used for 16S rRNA gene amplicon and shotgun metagenomic sequence analyses
**Table S2:** The quality and taxonomy of reference sequences of Candidatus Saccharimonadia recovered from various samples worldwide, including activated sludge samples
**Table S3:** The quality and taxonomy of the metagenomic bins of Candidatus Saccharimonadia obtained from size‐fractionated activated sludge samples
**Table S4:** Number of bins, genome size, completeness, contamination, and GC content of reconstructed bins of Candidatus Saccharimonadia from size‐fractionated activated sludge samples
**Table S5:** The number of ATPase genes by each bin belonging to order CAILAD01 based on Blast KEGG Orthology And Links Annotation (BlastKOALA)
**Table S6:** Sequence identity between Candidatus saccharimonadial sequences obtained from the metagenomic analysis using size‐fractionated activated sludge samples and sequences of pili genes retained by reference genomes
**Table S7:** Results of homology search between Candidatus saccharimonadial sequences obtained from the metagenomic analysis using size‐fractionated activated sludge samples and the type IV secretion system gene clusters of Candidatus Nanosynbacter lyticus TM7x. The numbers in the boxes indicate the locus tag of genes homologous to the related gene of Ca. Nanosynbacter lyticus strain TM7x
**Table S8:** Results of homology search between Candidatus saccharimonadial reference sequences and the type IV secretion system gene clusters of Candidatus Nanosynbacter lyticus TM7x. The numbers in the boxes indicate the locus tag of genes homologous to the related gene of Ca. Nanosynbacter lyticus strain TM7x.

**Data S1:** Supporting Information

## Data Availability

The data that support the findings of this study are openly available in DDBJ at https://www.ddbj.nig.ac.jp/index‐e.html, reference number PRJDB20466.
