# Supplementary material for: Metabolic Potential of Candidatus Saccharimonadia Including Rare Lineages in Activated Sludge
Source: Environ Microbiol Rep. 2025 Nov 6;17(6):e70231. doi: 10.1111/1758-2229.70231 (PMC12592800; doi:10.1111/1758-2229.70231)
Supplement: Supplementary file 1 — Figure S1: A genome‐based maximum likelihood phylogenetic tree of Candidatus Saccharimonadia. The reconstructed bins in this study are shown in bold and meticulously colour‐coded by the samples: purple for MGA, light blue for HAC, red for BEP, green for NRA, yellowish green for NRA_A2O, and yellow for YHG. A circle next to the bin's ID indicates the average nucleotide identity (ANI) value between the bins and the reference genomes in the genome taxonomy database r214: a black circle for an ANI value between 75 and less than 85, a grey circle for an ANI value between 85 and less than 95, and a white circle for an ANI value greater than 95. No circles are shown, indicating a bin for which it could not be calculated ANI values. Complete genomes are marked with a star. The names of the families for which genomes have not been recovered from activated sludge to date are shown in red. The isolation source and its location are shown in parentheses. Black circles at the nodes indicate bootstrap values of 95% or higher (1000 replicates) Figure S2: Completeness of the metabolic pathways of Candidatus Saccharimonadia reference genomes. “o_” indicates an order name; without “o_,” indicates a family name. Numbers in parentheses after the phylogenetic name indicates the number of reference genomes Figure S3: Phylogenetic tree based on genes homologous to effector genes of Candidatus Nanosynbacter lyticus strain TM7x. After the bin/reference genome names, the underscores indicate the effector gene locus tags. The letters' colours indicate that the red represents the Ca. Nanosynbacter lyticus TM7x strain, the blue represents the bin/reference genome of the order CAILAD01, and the grey represents the outgroup. Complete genomes are marked with a star. The numbers in parentheses (1–6) correspond to the effector gene locus tags of Ca. Nanosynbacter lyticus strain TM7x, TM7x_00090–TM7x_00100, indicating effector genes with homology (≤ 1e‐5 e‐value) to the genes in the bins/reference gen [file EMI4-17-e70231-s001.docx]

**– Supporting Information 1–**

**Metabolic Potential of *Candidatus* Saccharimonadia Including Rare Lineages in Activated Sludge**

Shuka Kagemasa^1,2,3^, Kyohei Kuroda^3*^, Ryosuke Nakai^3^, Mikiko Sato^4^, Yu-You Li^1,4^, Kengo Kubota^1,4*^

^1^Department of Civil and Environmental Engineering, Tohoku University, 6–6–06, Aza-Aoba, Aramaki, Aoba-ku, Sendai, Miyagi 980–8579, Japan

^2^Department of Creative Technology Engineering, National Institute of Technology, Anan College, 265 Aoki, Minobayashi-cho, Anan, Tokushima, 774-0017, Japan

^3^Biomanufacturing Process Research Center, National Institute of Advanced Industrial Science and Technology (AIST), 2‐17‐2‐1 Tsukisamu‐Higashi, Toyohira‐ku, Sapporo, Hokkaido, 062‐8517, Japan

^4^Department of Frontier Sciences for Advanced Environment, Tohoku University, 6–6–06, Aza-Aoba, Aramaki, Aoba-ku, Sendai, Miyagi 980–8579, Japan


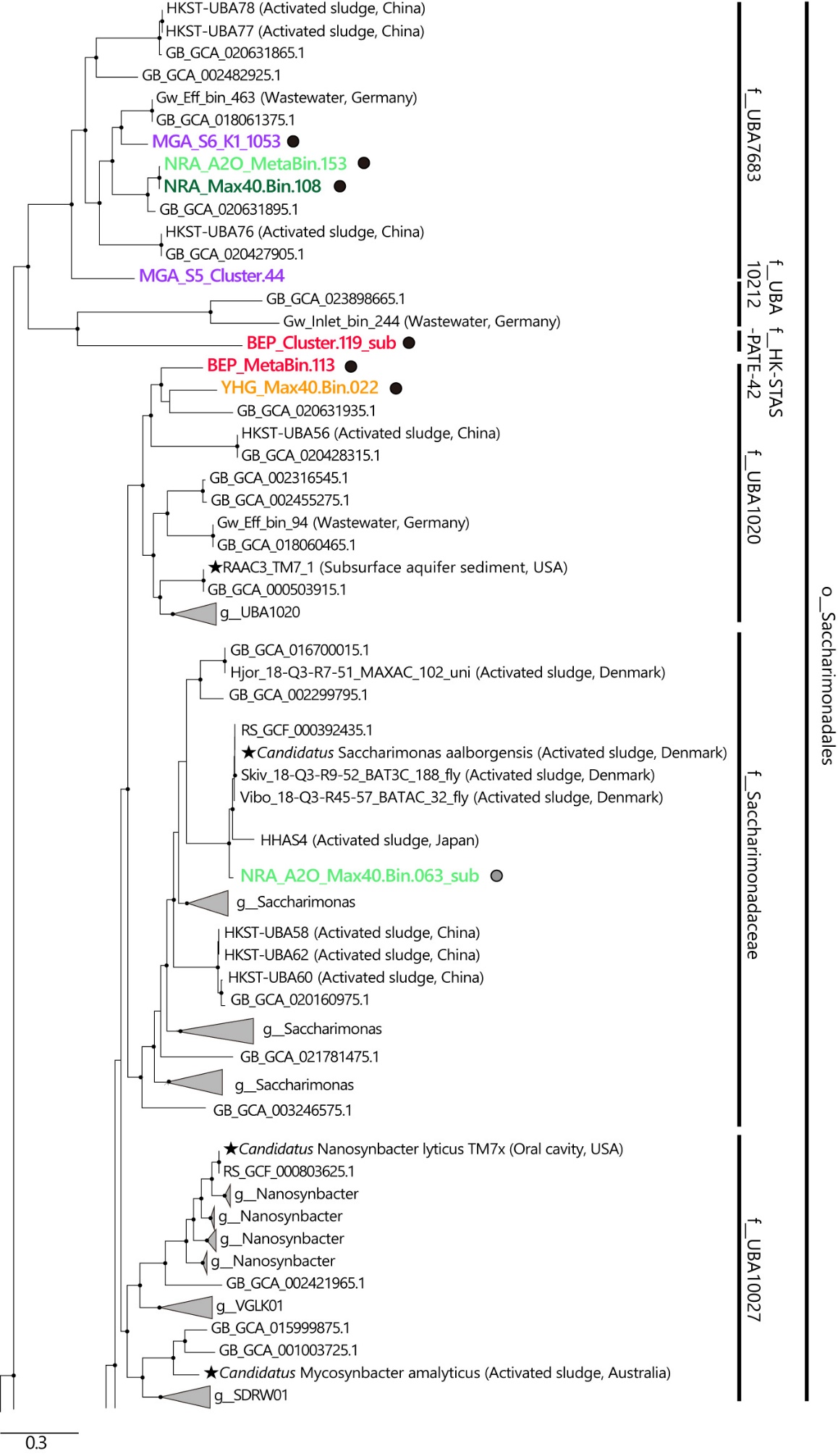


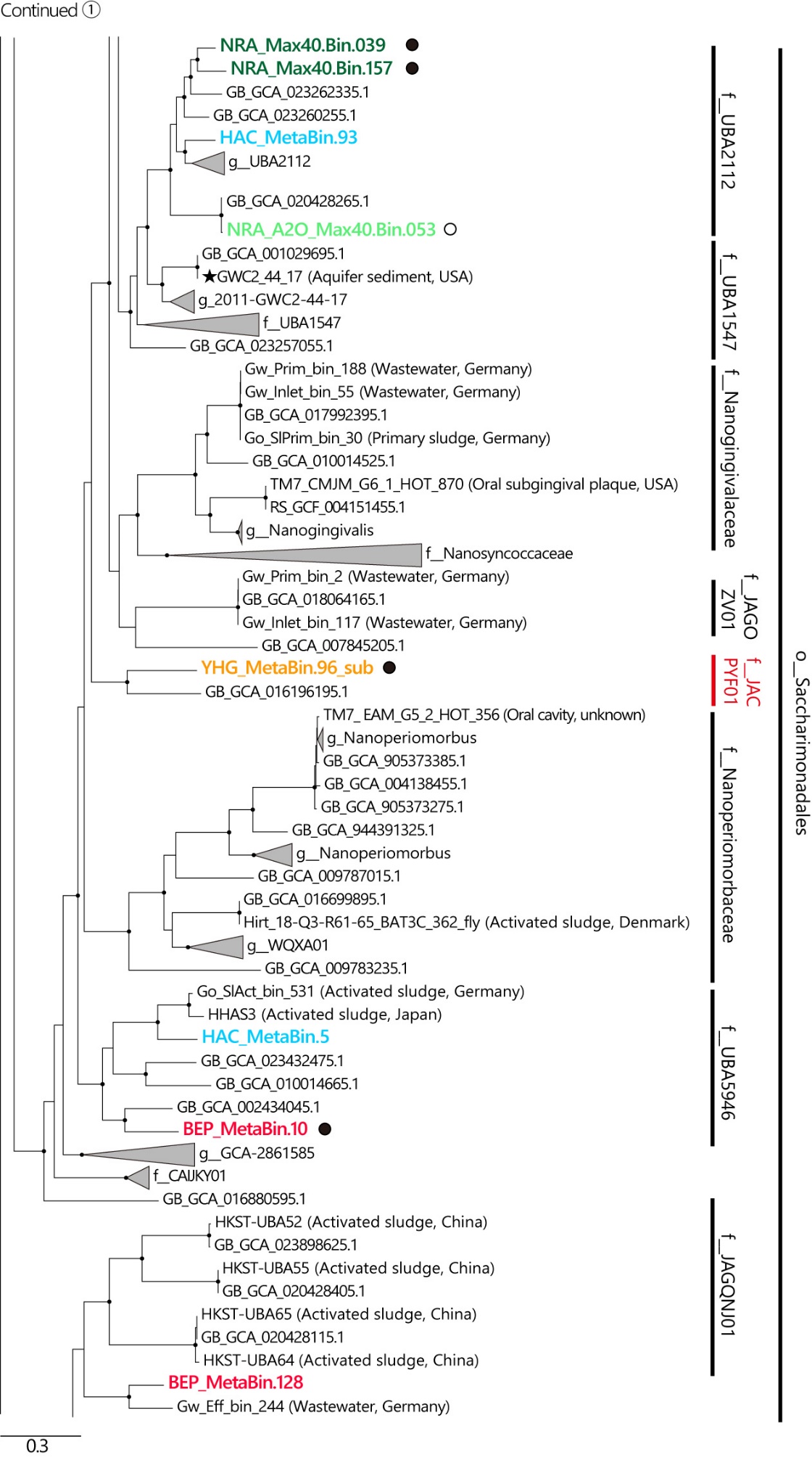


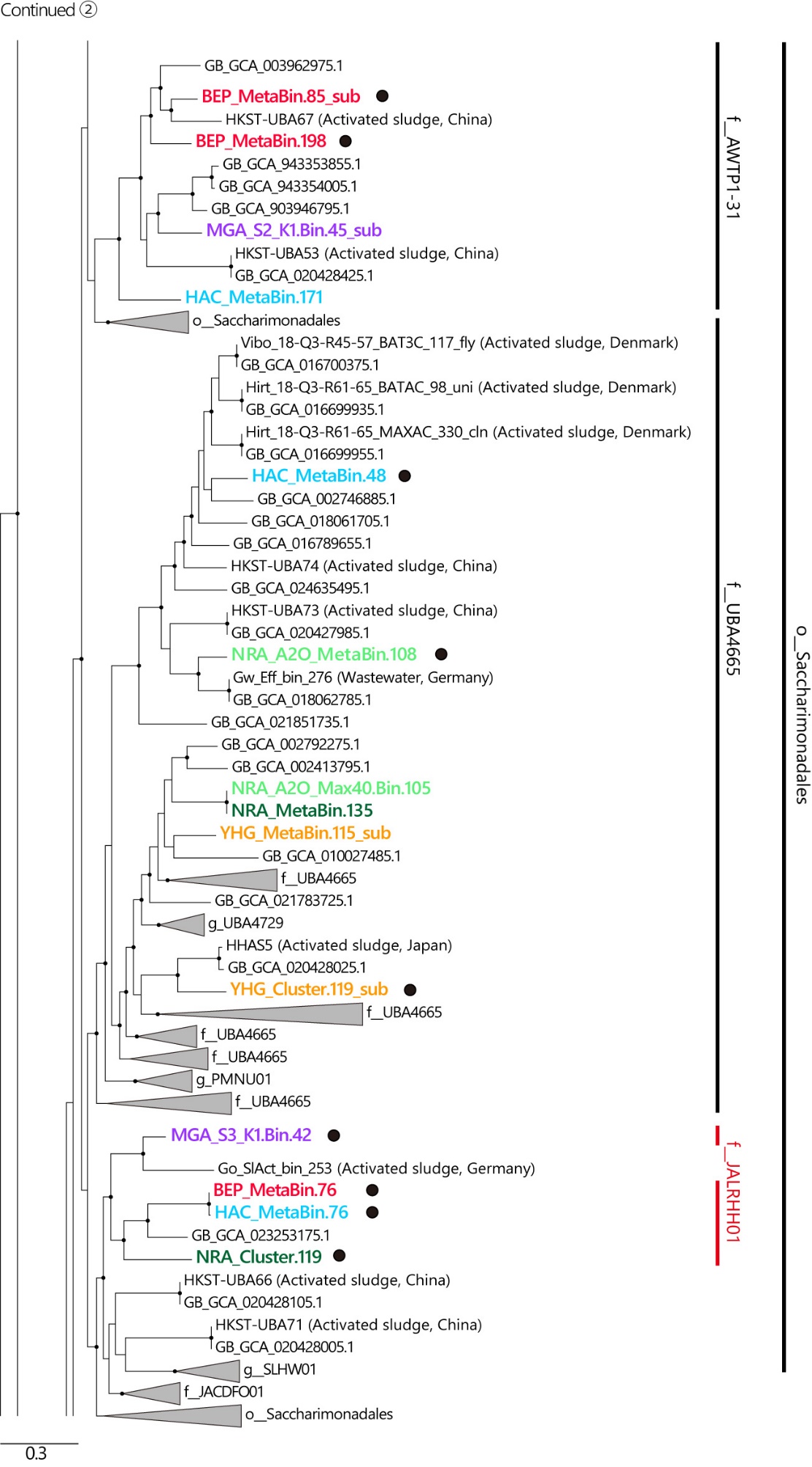


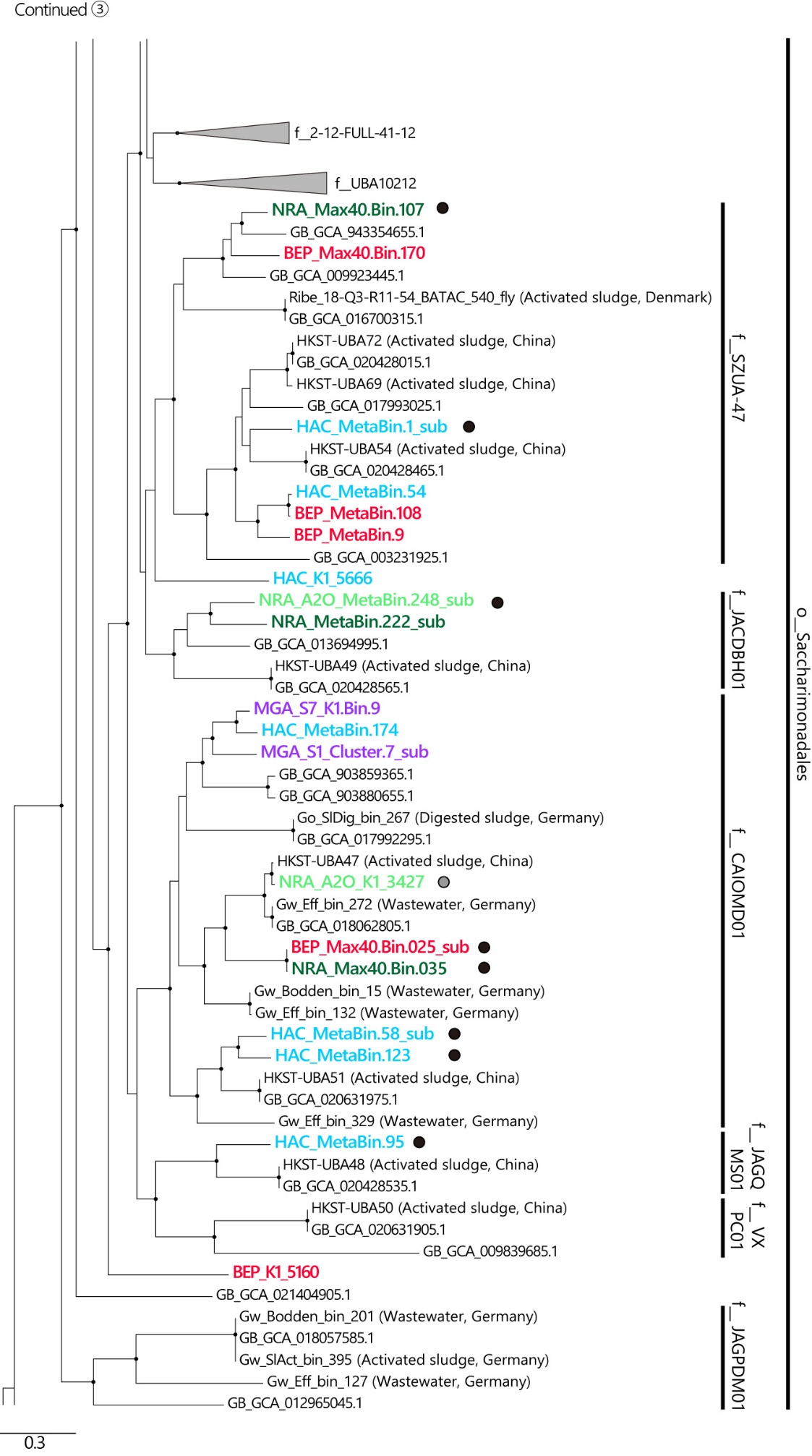


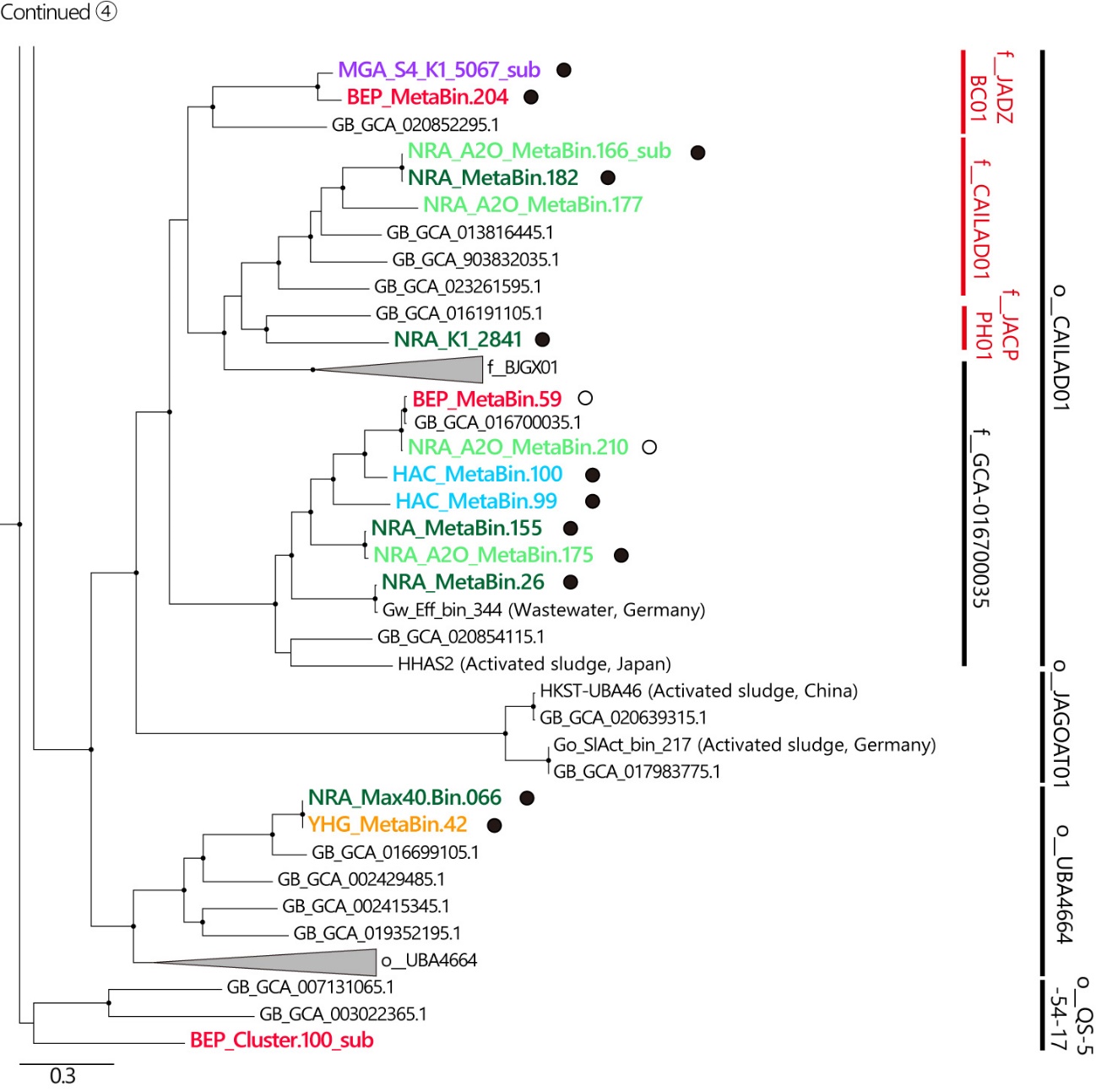


Fig. S1. A genome-based maximum likelihood phylogenetic tree of *Candidatus* Saccharimonadia. The reconstructed bins in this study are shown in bold and meticulously color-coded by the samples: purple for MGA, light blue for HAC, red for BEP, green for NRA, yellowish green for NRA_A2O, and yellow for YHG. A circle next to the bin's ID indicates the average nucleotide identity (ANI) value between the bins and the reference genomes in the genome taxonomy database r214: a black circle for an ANI value between 75 and less than 85, a grey circle for an ANI value between 85 and less than 95, and a white circle for an ANI value greater than 95. No circles are shown, indicating a bin for which it could not be calculated ANI values. Complete genomes are marked with a star. The names of the families for which genomes have not been recovered from activated sludge to date are shown in red. The isolation source and its location are shown in parentheses. Black circles at the nodes indicate bootstrap values of 95% or higher (1,000 replicates).

**Fig. S2. Completeness of the metabolic pathways of *Candidatus* Saccharimonadia reference genomes.** "o_" indicates an order name; without "o_," indicates a family name. Numbers in parentheses after the phylogenetic name indicates the number of reference genomes.


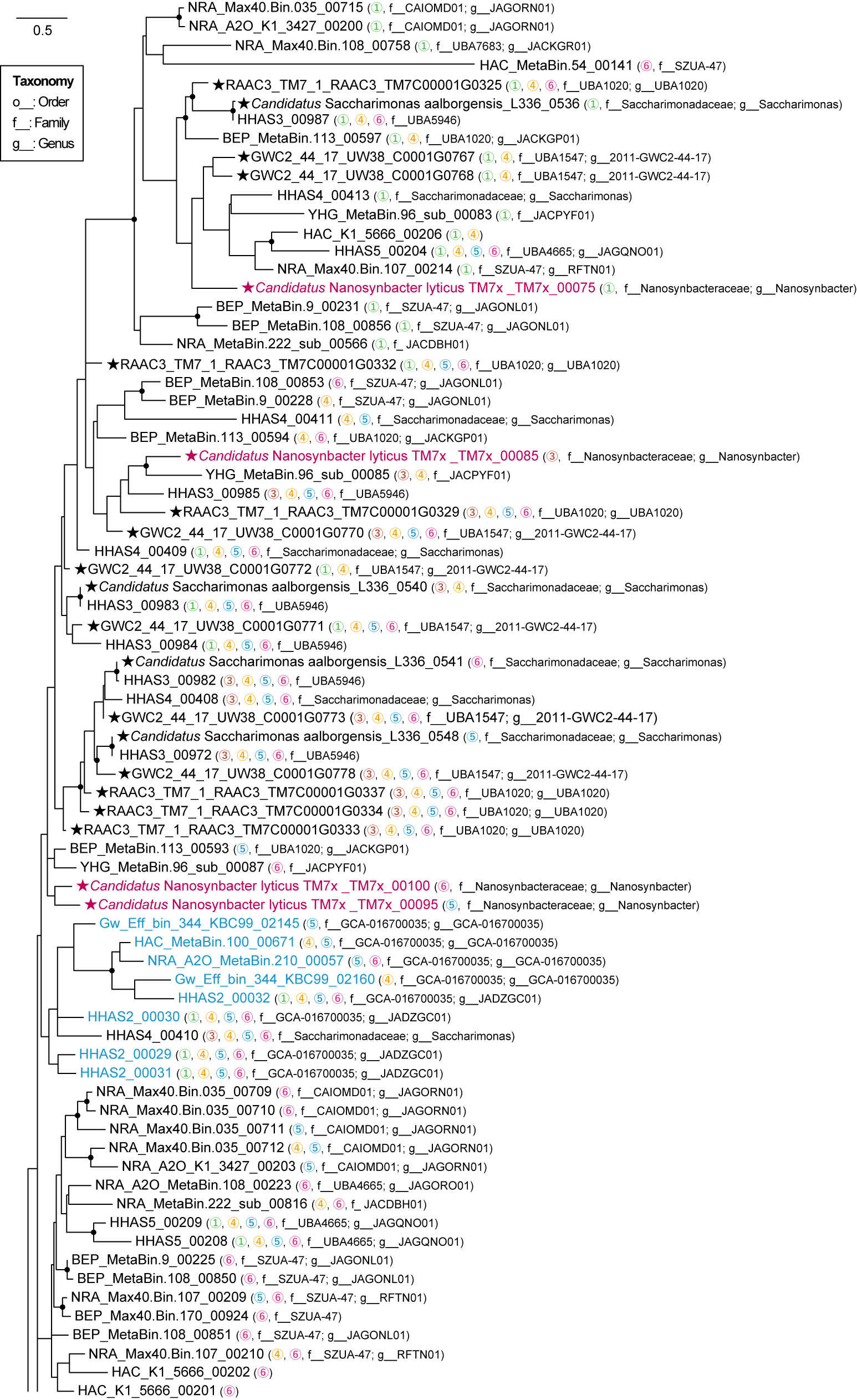

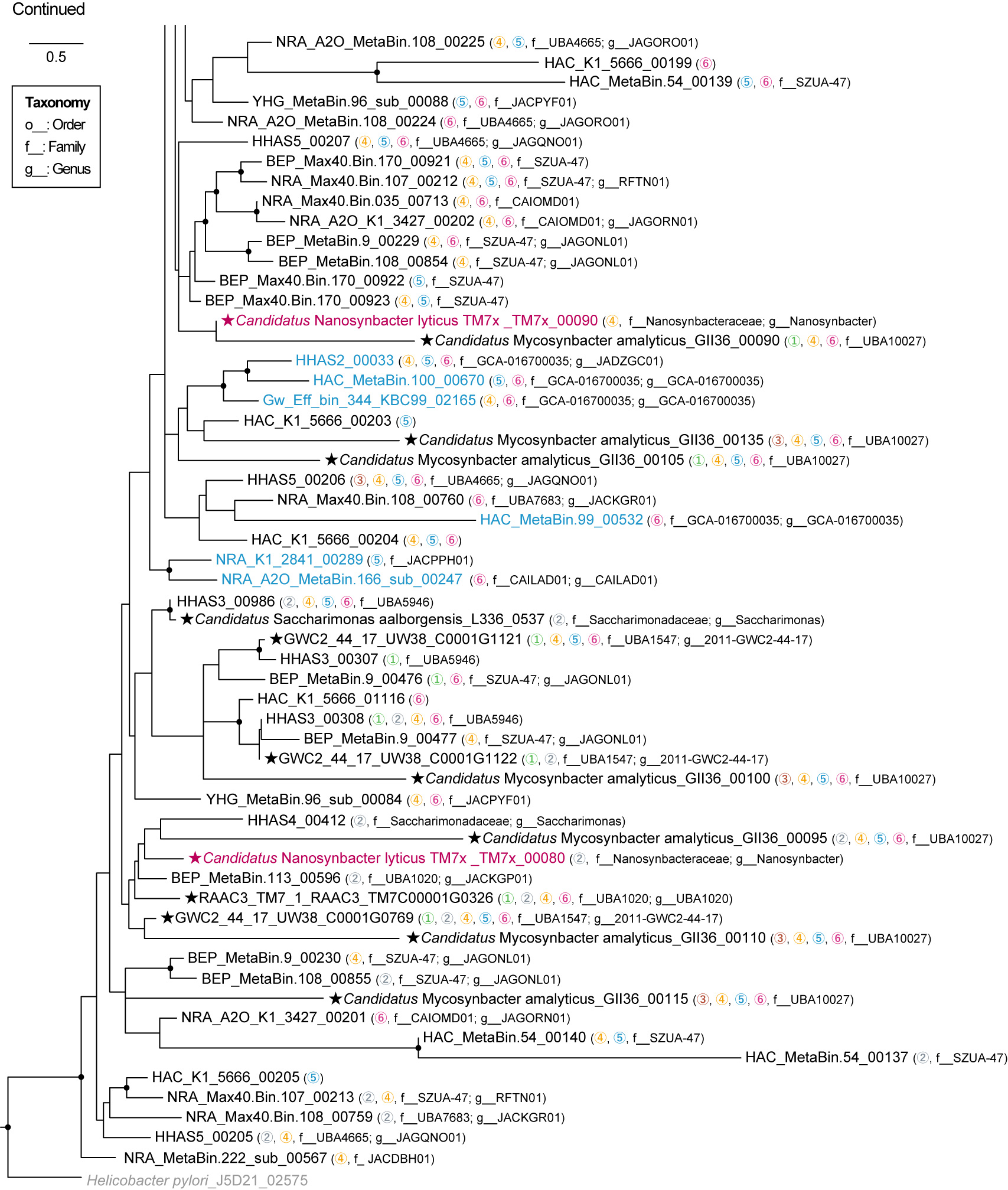


**Fig. S3. Phylogenetic tree based on genes homologous to effector genes of *Candidatus* Nanosynbacter lyticus strain TM7x.** After the bin/reference genome names, the underscores indicate the effector gene locus tags. The letters' colors indicate that the red represents the *Ca*. Nanosynbacter lyticus TM7x strain, the blue represents the bin/reference genome of the order CAILAD01, and the gray represents the outgroup. Complete genomes are marked with a star. The numbers in parentheses (1–6) correspond to the effector gene locus tags of *Ca*. Nanosynbacter lyticus strain TM7x, TM7x_00090–TM7x_00100, indicating effector genes with homology (≤1e-5 e-value) to the genes in the bins/reference genomes. The names of the lineages to which the bins/reference genomes to which the genes were detected belong are also shown in parentheses. Black circles at the nodes indicate bootstrap values of 95% or higher (1,000 replicates).
