# Supplementary material for: Metabolic Potential of Candidatus Saccharimonadia Including Rare Lineages in Activated Sludge
Source: Environ Microbiol Rep. 2025 Nov 6;17(6):e70231. doi: 10.1111/1758-2229.70231 (PMC12592800; doi:10.1111/1758-2229.70231)
Supplement: Supplementary file 3 — Data S1: Supporting Information [file EMI4-17-e70231-s002.docx]

**– Supporting Information 2–**

**Metabolic Potential of *Candidatus* Saccharimonadia Including Rare Llineages in Activated Sludge**

Shuka Kagemasa^1,2,3^, Kyohei Kuroda^3*^, Ryosuke Nakai^3^, Mikiko Sato^4^, Yu-You Li^1,4^, Kengo Kubota^1,4*^

^1^Department of Civil and Environmental Engineering, Tohoku University, 6–6–06, Aza-Aoba, Aramaki, Aoba-ku, Sendai, Miyagi 980–8579, Japan

^2^Department of Creative Technology Engineering, National Institute of Technology, Anan College, 265 Aoki, Minobayashi-cho, Anan, Tokushima, 774-0017, Japan

^3^Biomanufacturing Process Research Center, National Institute of Advanced Industrial Science and Technology (AIST), 2‐17‐2‐1 Tsukisamu‐Higashi, Toyohira‐ku, Sapporo, Hokkaido, 062‐8517, Japan

^4^Department of Frontier Sciences for Advanced Environment, Tohoku University, 6–6–06, Aza-Aoba, Aramaki, Aoba-ku, Sendai, Miyagi 980–8579, Japan

**Methods of construction phylogenetic trees based on effector genes**

***Selection of bins recovered from size-fractionated activated sludge and reference genomes for constructing a phylogenetic tree based on effector genes.***

The construction of phylogenetic trees was based on amino acid sequences for effectors. The phylogenetic tree was constructed using the amino acid sequences of the obtained bins (*n* = 18, Table S7) from fractionated samples and the reference genome (*n* = 9, Table S8), which may retain all the genes of *virB4*, *virB6*, *virB8*, and effector cluster. *virB4*, *virB6*, and *virB8* are type IV secretion system (T4SS) genes that may be retained in over half of the bins recovered from the fractionated samples. We determined that the obtained bins/reference genomes retained the effector cluster based on a set of criteria. These criteria include three or more consecutive locus tags for effector genes or four or more consecutive locus tags for effector genes, including one missing gene in the middle of the gene cluster. However, the complete genomes (*n* = 5, Table S8 (Albertsen *et al*., 2013; Batinovic *et al*., 2021; Brown *et al*., 2015; He *et al*., 2015; Kantor *et al*., 2013)) were used for phylogenetic tree construction regardless of the genome selection criteria described above. Although no bins/genomes belonging to the order CAILAD01 retained effector cluster, these bins/genomes may have retained effector genes. Therefore, the bins/genomes belonging to the order CAILAD01, which may retain all of the *virB4*, *virB6*, and *virB8* genes and effector genes (Tables S7 and S8), were used to construct a phylogenetic tree. *Helicobacter pylori* (GCA_017821535.1), which retains the genes for T4SS, was selected as an outgroup in the phylogenetic tree.

***Construction phylogenetic trees based on effector genes***

Amino acid sequences of the effector genes in selected bins/reference genomes were aligned using mafft ver. 7.520 (Katoh *et al*., 2002) and trimAl ver. 1.4.1. (Capella-Gutiérrez *et al*., 2009). The phylogenetic tree was constructed using iqtree2 version 2.1.2, with automatically optimized substitution models of LG+G4. The phylogenetic tree numbers of bootstrap and SH-like approximate likelihood ratio test replicate counts were specified to be 1000 each.

**References**

Albertsen, M., Hugenholtz, P., Skarshewski, A., Nielsen, K.L., Tyson, G.W., and Nielsen, P.H. (2013). Genome sequences of rare, uncultured bacteria obtained by differential coverage binning of multiple metagenomes. *Nature biotechnology* 31 (6), 533–538. https://doi.org/10.1038/nbt.2579

Batinovic, S., Rose, J.J., Ratcliffe, J., Seviour, R.J., and Petrovski, S. (2021). Cocultivation of an ultrasmall environmental parasitic bacterium with lytic ability against bacteria associated with wastewater foams. *Nature microbiology* 6 (6), 703–711. https://doi.org/10.1038/s41564-021-00892-1

Brown, C.T., Hug, L.A., Thomas, B.C., Sharon, I., Castelle, C.J., Singh, A., Wilkins, M.J., Wrighton, K.C., Williams, K.H., and Banfield, J.F. (2015). Unusual biology across a group comprising more than 15% of domain Bacteria. *Nature* 523 (7559), 208–211. https://doi.org/10.1038/nature14486

Capella-Gutiérrez, S., Silla-Martínez, J. M., and Gabaldón, T. (2009). trimAl: a tool for automated alignment trimming in large-scale phylogenetic analyses. *Bioinformatics* 25 (15), 1972–1973. https://doi.org/10.1093/bioinformatics/btp348

He, X., McLean, J.S., Edlund, A., Yooseph, S., Hall, A.P., Liu, S.Y., Dorrestein, P.C., Esquenazi, E., Hunter, R.C., Cheng, G., Nelson, K.E., Lux, R., and Shi, W. (2015). Cultivation of a human-associated TM7 phylotype reveals a reduced genome and epibiotic parasitic lifestyle. *Proceedings of the National Academy of Sciences of the United States of America* 112 (1), 244–249. https://doi.org/10.1073/pnas.1419038112

Kantor, S.R., Wrighton, C.K., Handley, M.K., Sharon, I., Hug, A. L., Castelle, J.C., Thomas, C.B., and Banfield, F.J. (2013). Small genomes and sparse metabolisms of sediment-associated bacteria from four candidate phyla. *mBio* 4 (5), e00708–e00713. https://doi.org/10.1128/mBio.00708-13

Katoh, K., Misawa, K., Kuma, K., and Miyata, T. (2002). MAFFT: a novel method for rapid multiple sequence alignment based on fast Fourier transform. *Nucleic acids research* 30 (14), 3059–3066. https://doi.org/10.1093/nar/gkf436
